# Effectiveness of vapocoolant spray compared to eutectic lidocaine/prilocaine cream to enhance tolerance during intravenous catheterisation: a randomised controlled trial

**DOI:** 10.1111/jsap.13825

**Published:** 2025-01-13

**Authors:** R. Trinder, J. Park, K. Humm, L. Cole

**Affiliations:** ^1^ Department of Clinical Science and Services The Royal Veterinary College, University of London Hatfield UK; ^2^ Small Animal Teaching Hospital, Institute of Veterinary Science, University of Liverpool Liverpool UK

## Abstract

**Objectives:**

To determine if tolerance of intravenous catheterisation differs following the application of vapocoolant spray compared to lidocaine/prilocaine cream in dogs and cats.

**Materials and Methods:**

A randomised controlled trial of client‐owned dogs and cats requiring intravenous catheterisation was performed. They were randomly allocated to either have lidocaine/prilocaine cream applied to their skin 1 hour prior to intravenous catheterisation or a swab saturated with vapocoolant spray applied immediately prior to intravenous catheterisation. The procedure was video‐recorded and a single blinded observer reviewed the recordings and assigned reaction scores (0 to 3) at 4 time points (initial restraint, limb handling, swab application and skin puncture).

**Results:**

Between October 2020 and March 2022, a total of 101 animals (83 dogs and 18 cats) were enrolled, with 56 patients randomised to receive vapocoolant spray and 45 to receive lidocaine/prilocaine cream. There was no significant difference in the age, sex status, number of cross and pure breeds, and mentation detected between the groups. There was no significant difference in reaction scores between the treatments when comparing all patients at any time point except for a significantly increased swab application reaction score in patients receiving vapocoolant spray. Vapocoolant spray was significantly less effective in reducing adverse reaction to skin puncture than lidocaine/prilocaine cream in the small number of cats evaluated.

**Clinical Significance:**

When considering all patients together, no single method of anaesthesia appeared superior for improving tolerance of intravenous catheter placement. However, vapocoolant spray may be less effective than lidocaine/prilocaine cream in reducing adverse response to skin puncture during catheterisation in cats.

## INTRODUCTION

Venepuncture for blood sampling and intravenous catheter placement is a necessary and unavoidable treatment for many veterinary patients; however, it can often be associated with significant stress and potential discomfort (Chebroux et al., 2015). Although the degree of discomfort is generally accepted as minimal, any degree of pain could result in aversive responses by patients and potentially make the procedure more difficult.

Eutectic mixture of local anaesthetics (EMLA) cream is a topical anaesthetic product containing 2.5% lidocaine and 2.5% prilocaine and has been widely demonstrated to reduce discomfort and distress in humans undergoing intravenous catheterisation (IC) (Bond et al., 2016). Similarly, in veterinary literature EMLA has been demonstrated to reduce discomfort and distress of patients during venepuncture and peripheral and central IC (Crisi et al., 2020; Flecknell et al., 1990; van Oostrom & Knowles, 2018; Wagner et al., 2006). Despite its demonstrated benefits, EMLA cream has some disadvantages compared to IC without topical anaesthetic products such as additional cost, and the delay of 60 minutes from application to optimal effect (Gibbon et al., 2003; van Oostrom & Knowles, 2018; Wagner et al., 2006).

Vapocoolant sprays (VS) are a class of cryoanaesthetics that are described to provide rapid anaesthetic effects. The VS contains a volatile liquid (commonly ethyl chloride) that rapidly evaporates once applied to the skin, effectively lowering surface temperatures (Lomax et al., 2017, 2018). This rapid reduction in temperature reduces the rate of nerve conduction in a linear manner until 10°C, at which point neural transmission and receptor sensitivity is effectively impeded, including nociception, allowing immediate anaesthetic effects (Denny‐Brown et al., 1945; Kunesch et al., 1987; Millis, 2004; Paintal, 1965). The application of VS has been assessed in several randomised controlled trials and meta‐analyses for its ability to effectively reduce discomfort of venepuncture and IC in adults and children (Barbour et al., 2018; Mace, 2016; Zhu et al., 2018). However, by contrast there is limited research in the veterinary literature examining the analgesic effects of VS, with a small number of large animal studies having demonstrated its efficacy in reducing pain associated with a variety of minor procedures, including ear notching, ear tagging and intra‐articular injection (Fjordbakk & Haga, 2011; Lomax et al., 2017, 2018; Van Der Saag et al., 2019). However, in the only study examining the application of VS in canine and feline patients, VS failed to demonstrate a significant reduction in adverse responses to IC when compared to a saline control in patients undergoing IC (Trinder et al., 2022).

Direct comparison between the use of EMLA and VS has been examined in a handful of publications in the human literature, with mixed results. In a crossover study of 40 children, VS was not found to be as effective as EMLA cream at reducing pain associated with IC (Dalvandi et al., 2017). This was supported by a two‐period randomised cross‐over trial of adult volunteers who associated EMLA cream with reduced pain, and greater satisfaction when compared to VS (Thind et al., 2021). Whilst in a study of children receiving intramuscular vaccination, no significant difference in discomfort and behavioural changes were noted between patients receiving EMLA or VS (Cohen Reis & Holubkov, 1997). In the veterinary literature, only a single study has directly compared the effects of VS and EMLA cream; in kid goats undergoing horn debudding, and this demonstrated no significant difference in pain response between the populations (Cuttance et al., 2022). Searches of internet databases [Medline (PubMed), Ovid and Google Scholar] were unable to demonstrate any further reports of comparisons between VS and EMLA in the veterinary literature.

The primary aim of this study was to examine if there was a difference in tolerance of IC following the application of VS when compared to EMLA. Our secondary aim was to determine if either topical application technique significantly improved facility to IC access. We hypothesised that there would be no significant difference in patient reaction or IC success between those receiving VS and those receiving EMLA.

## MATERIALS AND METHODS

This blinded randomised controlled trial prospectively enrolled dogs and cats presenting for either blood donation collection (cats only), or as new oncology patients (cats and dogs) to a referral hospital. This population was chosen as they routinely receive EMLA prior to IC and, as such, inclusion in the study would not prolong their hospitalisation time. The placement of IC in blood donors is only routinely performed in feline donors at our institution. Written client consent was obtained prior to enrolment with ethical approval granted by the institution's ethics and welfare committee (URN 2020 1998‐3). Patients were excluded if client consent was not obtained, or were perceived to require sedation prior to IC attempt.

Recruited patients had their breed, sex status, service (donor or oncology), and age recorded as well as a previously described mentation score (Hayes et al., 2010), which was determined by the supervising clinician/registered veterinary nurse at the time of enrolment immediately prior to catheter placement.

At the time of enrolment into the study patients were randomised to either receive VS (Ethycalm, Invicta Animal Health Ltd) or lidocaine/prilocaine cream (EMLA™ Cream, AstraZeneca) (EMLA), using an internet‐based randomisation tool (Sealed Envelope, Sealed Envelope Ltd, UK). A patient could only be enrolled once, with the treatment and recording only occurring on their first catheterisation attempt.

The intravenous catheter placement site was either cephalic, lateral or medial saphenous vein (determined by the person placing the catheter). Following randomisation, a small area of fur over the vein was clipped, with those patients randomised to receive EMLA having approximately 1.5 g of the cream applied topically to the area and covered with non‐absorbent plastic 60 minutes prior to IC attempt, as previously described (van Oostrom & Knowles, 2018). Those patients randomised to receive VS had the site of catheterisation prepared in an identical manner, however, had no EMLA cream was applied prior to the application of the non‐absorbent plastic. The catheter size selected by the placer was ultimately at their discretion with the guiding principle of the largest gauge catheter that could be placed safely and confidently in prepared vein being selected.

The process of IC was video recorded from the point of initial patient restraint until placement of the catheter, with the recorder and placer non‐blinded to the group allocation. Restraint was provided in all circumstances by trained registered veterinary nurses, in a standardised manner in accordance with institution and international guidelines (Chapman, 2017; Rodan et al., 2022). Placement protocol was standardised, with the non‐absorbent plastic removed from the skin then aseptically prepared using a chlorhexidine gluconate solution (Chloraprep, BD). The patients receiving VS had the prepared area wiped four times with a swab that had been soaked with VS for a duration of at least 4 seconds. The patients receiving the EMLA had a sterile saline‐soaked swab applied as an alternative, to mimic the four wipes of the patients receiving VS. Both groups then had a single wipe from an isopropyl alcohol swab prior to the IC attempted.

All recordings were sound and video edited to remove any indication of potential treatment and were subsequently reviewed by a single blinded observer (LC). The observer assigned reaction scores (0 to 3) at 4 time points; initial restraint, touch of the limb by the person placing the catheter, application of treatment or control swab and when the skin was punctured by the catheter using previously described scoring systems (Tables 1 and 2) (Crisi et al., 2020; Flecknell et al., 1990; Gibbon et al., 2003; Trinder et al., 2022; van Oostrom & Knowles, 2018; Wagner et al., 2006).

**Table 1:** Restraint reaction scoring system for assessment of animal compliance during restraint for catheterisation

Criteria	Observation	Score
Restraint – struggling	None	0
	Mild (tense body)	1
	Moderate (struggle)	2
	Severe (escape restraint)	3
Restraint – aggression	None	0
	Mild (hisses/snarl)	1
	Moderate (attempt to scratch)	2
	Severe (attempt to bite)	3

**Table 2:** Reaction scoring system for assessment of animal response to their limb being handled, swab application and skin puncture during catheterisation

Observation	Score
No reaction	0
Slight movement of limb, tensing of muscles	1
Limb withdrawal, attempting to move away	2
Marked attempts to escape, aggressive behaviour, vocalisation	3

Additional information was recorded with each IC attempt including who had attempted to place the catheter (student, nurse or veterinarian), whether the IC attempt was successful, and whether the patient later required sedation for future IC attempts. A successful IC attempt was confirmed by performing a saline flush to demonstrate adequate flow whilst observing for the absence of swelling or oedema around the insertion site and was ultimately determined by the supervising clinician. The site where the skin was prepared following application of either product was also examined for evidence of an adverse reaction and recorded if evident.

### Sample size calculation

Data comparing the use of VS and EMLA in the reduction of discomfort of children in response to IC was used to calculate the sample size for this study (Dalvandi et al., 2017). Based on calculations using a commercial statistical programme (Epi Info™, CDC, USA), 50 animals per group were required to detect a four‐fold decrease in odds ratio (OR) of response to catheterisation in the patients receiving VS compared to EMLA, with 90% power and a 5% type I error rate.

### Statistical analysis

All statistical comparisons were performed using SPSS (IBM Corp., Armonk, USA). Data was tested for normality using a Shapiro–Wilk test. Normally distributed data was presented as mean ± SD and non‐normally distributed data expressed as median (IQR and range). An unpaired *t*‐test was used to compare normally distributed continuous data, a Mann–Whitney *U* test was used to compare non‐normally distributed ordinal data, with P‐values adjusted using a Bonferroni correction, and chi‐squared, Fischer's exact test or binomial tests were used to assess categorical data. An ordinal uni‐ and multivariable logistic regression analysis was performed to evaluate the association of individual variables (treatment – VS/EMLA, sex, mentation, placer, species, age and service) with each reaction score. Variables with P‐values <0.25, with a minimum of 10 subjects were included in multivariable analysis. Significance was determined as a P‐value <0.05.

## RESULTS

A total of 120 animals were recruited between October 2020 and March 2022 with one excluded for failure to comply with standardised catheterisation protocol, whilst a further 18 were lost following enrolment due to lack of progression to IC or lack of recording of the IC process. Therefore 101 animals were enrolled; 83 dogs and 18 cats with a mean age of 8.62 years (SD ± 3.16) (Table 3).

**Table 3:** Demographic data of 83 dogs and 18 cats enrolled into the study

Groups	Vapocoolant spray	EMLA	P‐value	
Total, *n*	56	45	0.32	
Species, *n*				0.037
Dog	50	33	0.08	
Cat	6	12	0.24	
Age, years	8.71 ± 3.00	8.49 ± 3.36	0.91	
Sex status, *n*			0.898	
Male neutered	27	22		
Male entire	6	4		
Female neutered	21	16		
Female entire	2	3		
Breed			0.534	
Pure breed	46	39		
Cross breed	10	6		
Mentation (Hayes et al., 2010), *n*			0.67	
0	55	43		
1	1	2		
2	0	0		
3	0	0		

EMLA Eutectic lidocaine/prilocaine anaesthetic

Fifty‐six patients were randomised to receive VS (50 dogs and six cats) and 45 to receive EMLA (33 dogs and 12 cats) with no statistical difference in baseline characteristics; age, breed, sex and mentation. There was no significant difference in the number of patients receiving each treatment when compared for all patients, dogs or cats; however, a significantly greater number of cats receiving EMLA compared to those receiving VS when compared to dogs (P = 0.037) (Table 3). A total number of 96 intravenous catheter attempts were performed in the forelimbs, whilst only six were attempted in the hindlimb with no significant difference between the two groups (P = 1.00).

Thirteen of the patients were feline blood donations (VS 5, EMLA 8), whilst the remaining 88 patients were oncological patients (VS 51, EMLA 37), consisting of 83 dogs and 5 cats.

Of the 101 recorded intravenous catheter attempts, 52 were performed by students (VS 31, EMLA 21) and 49 by nurses (VS 25, EMLA 24) with no significant difference between those patients that received VS and those that received EMLA (P = 0.39).

### Reaction scores

When comparing reactions scores between those patients that received VS and those that received EMLA, no significant difference was detected when assessing patient response to restraint, limb handling or skin puncture with insufficient variation in score to compare response to restraint (aggression) (Table 4). However, those patients receiving VS had greater than 6 and half times the odds of showing an increased reaction score during swab application compared to patients receiving EMLA [95% confidence interval (CI) 2.06 to 20.64, P = 0.001] (Table 4). Univariable analysis demonstrated no significant difference in mentation, placer, species, age or service in relation to the response to catheterisation (Table 5). Only treatment met the threshold for multivariable analysis, and alone did not meet significance.

**Table 4:** Univariable ordinal logistical regression results for the association between treatment groups and response at each observed time point on a response scale from 0 (no response) to 3 (severe response)

Variable	Category	OR	95% CI	P‐valve
Restraint – aggression	VS	N/A†	N/A†	N/A†
	EMLA	1	–	–
Restraint – struggling	VS	2.17	0.01 to 11.15	0.40
	EMLA	1	–	–
Limb touch	VS	2.172	0.71 to 6.69	0.18
	EMLA		–	
Wipe	VS	6.52	2.06 to 20.64	0.001
	EMLA	1	–	–
Skin puncture	VS	1.91	0.86 to 4.25	0.11
	EMLA	1	–	–

VS Vapocoolant spray, EMLA Eutectic lidocaine/prilocaine anaesthetic, OR Odds ratio, CI Confidence interval, N/A Not applicable

^†^ Insufficient events to be statistically evaluated

**Table 5:** Univariable ordinal logistical regression results for the association between independent variables and response to intravenous catheterisation scored on a response scale from 0 (no response) to 3 (severe response) in 83 dogs and 18 cats

Variable	Category	OR	95% CI	P‐valve
Treatment	VS	1.9	0.86 to 4.25	0.11
	EMLA	1	–	–
Sex	MN	2.61	0.07 to 2.06	0.26
	ME	0.25	0.03 to 1.90	0.18
	FS	0.12	0.02 to 0.03	0.19
	FE	1	–	–
Mentation	0	1.59	0.13 to 18.88	0.71
	1	–	–	–
Placer	Student	0.71	0.33 to 1.54	0.39
	Nurse	–	–	–
Species	Dog	1.01	0.37 to 2.78	0.98
	Cat	1	–	–
Age	Years	0.98	0.87 to 1.11	0.74
Service	Oncology	1.09	0.34 to 3.5	0.88
	Donor	1	–	–

VS Vapocoolant spray, EMLA Eutectic lidocaine/prilocaine anaesthetic, MN Male neutered, ME Male entire, FN Female neutered, FE Female entire, OR Odds ratio, CI Confidence interval

When examining just dogs' responses to VS or EMLA, no significant difference was detected when assessing their response to restraint (struggle – OR: 3.429, 95% CI: 0.39 to 30.27, P = 0.27), limb handling (OR 2.201, 95% CI: 0.65 to 7.46, P = 0.21) or skin puncture (OR 1.34, 95% CI: 0.56 to 3.24, P = 0.514). However, those canine patients receiving VS had approximately seven times the odds of showing an increased response during swab application (OR: 6.74, 95% CI 1.81 to 25.03, P = 0.004) compared to those receiving EMLA. By contrast, the comparison of reaction scores in cats demonstrated no statistically significant difference in response to swab application (OR: 4.38, 95% CI: 0.31 to 6.13, P = 0.27), with insufficient variation in scores to assess reason to restraint and limb holding. When assessing response to skin puncture, feline patients receiving VS had approximately 13 times the odds of showing an increased reaction to skin puncture (OR: 13.49, 95% CI: 1.44 to 126.85, P = 0.023).

The instance of struggle, aggression and reactions scores for all patients and specific species has been displayed in Tables S1 to S3.

No adverse skin reactions were reported in any of the patients, with three patients that were presented to the oncology service requiring sedation following catheterisation attempt (VS: 1, EMLA: 2).

### Intravenous catheter success

Successful IC was reported in 64% (65/101) animals of which 64% (36/56) placements were successful for those patients' receiving VS and 64% (29/45) were successful in those patients receiving EMLA, this was not significantly different (OR: 0.99, 95% CI 0.44 to 2.25, P = 0.99).

## DISCUSSION

The results of this study demonstrated no significant differences in the reaction scores of patients to IC when prior treated with VS or ELMA. These results differ from similar studies in humans where the application of EMLA was associated with either less severe, or more effective reduction in pain for arteriovenous fistula cannulation, and IC (Çelik et al., 2011; Dalvandi et al., 2017; Thind et al., 2021). However, when comparing the two treatments for minimally invasive procedures such as intramuscular injections and spinal injections no such difference is evident (Cohen Reis & Holubkov, 1997; Firdaus et al., 2018; Gupta et al., 2017). When examining the veterinary literature, our findings are similar to those described in a population of kid goats undergoing dehorning, in which no significant difference between the analgesic properties provided by either treatment was demonstrated (Cuttance et al., 2022). Given the heterogenous nature of these study populations, variation in topical anaesthetic application technique, and species differences, caution should be applied when comparing these findings to those presented in this study. Based on the literature search performed, there are no published reports of comparisons between the application of EMLA and VS in small animal veterinary species.

The application of EMLA in companion animals for the purposes of IC and venepuncture has been widely demonstrated to reduce adverse reaction of patients compared to control groups (Crisi et al., 2020; Flecknell et al., 1990; van Oostrom & Knowles, 2018; Wagner et al., 2006). VS, despite demonstrating a reduction in adverse response to minor procedures in larger animals (horses and cattle) (Fjordbakk & Haga, 2011; Lomax et al., 2017, 2018), failed to significantly reduce adverse response to IC in a population of emergency companion animals when compared to a control group administered no topical anaesthetic (Trinder et al., 2022).

The present study demonstrated a greater adverse response to the application of VS than a saline‐soaked swab when assessed in both cats and dogs. When divided by species, this greater adverse response was only present in the canine patients. This adverse response, and species variation, was similarly evident in the previous study of VS in canine and feline patients (Trinder et al., 2022). As in that study, this adverse response can likely be attributed to the coldness of the swab following VS application compared to saline control. Similarly, discomfort is reported upon application in a study of children, in which the VS had been applied to a cotton ball (Cohen et al., 2009).

Interestingly, when comparisons between reaction to skin puncture are separated by species feline patients receiving VS had approximately 13 times the odds of showing an increased reaction to skin puncture compared to those receiving EMLA. This would suggest in the feline patients examined in this study, VS was less effective at providing topical anaesthesia for the purposes of IC. In the previous study examining the use of VS in cats, its application was considered statistically not different to a saline control (Trinder et al., 2022). Caution should be taken however when interpreting these findings given the significantly greater number of cats receiving ELMA compared to dogs (P = 0.037), and a significantly greater number of cats receiving EMLA from the blood donor group, and the low number of animals in each group (VS = 6, EMLA = 12), as well as the overall low number of cats enrolled (18 of 101). Post‐hoc power calculation, with a 90% power, and a 5% type 1 error rate demonstrated a total of 48 dogs and 68 cats would be required per treatment group to detect a significant difference in reaction between individuals in each species group. In light of this, caution should be taken when interrupting species‐specific findings given the study is underpowered to effectively evaluate patient response when the species are considered separately.

No significant difference was recorded in IC success when comparing the use of VS or ELMA. The use of topical anaesthetics in veterinarian and human publications has found similar findings with no improvement in catheterisation success (Trinder et al., 2022; van Oostrom & Knowles, 2018; Zhu et al., 2018). When examining the category of placer (student and nurse), no significant difference in IC success was found between those patients receiving VS and ELMA. Experience level has been examined before in the veterinary literature when using topical anaesthetics for IC, with similar findings (Trinder et al., 2022; van Oostrom & Knowles, 2018).

No adverse skin reactions were reported as a result of the application of VS or ELMA. Similar results have been found in human and veterinary studies using a variety of VS application techniques (Fjordbakk & Haga, 2011; Trinder et al., 2022; Zhu et al., 2018). Significant tissue injury can occur secondary to cryoanaesthesia; however, they are typically reported when tissue temperatures reach below −20°C (Evans et al., 1981). EMLA is well tolerated in human patients with only minor adverse effects when used for the purpose local anaesthesia prior to IC and venepuncture such as reports of local blanching or erythema (Lener et al., 1997). In two studies specifically examining adverse effects of EMLA application in healthy and critically ill cats, no adverse topical or systemic complications were reported (Gibbon et al., 2003; Wagner et al., 2006). Whilst the only canine study examining the use of EMLA made no comment as to adverse effects of EMLA application for either 30 or 60 minute duration for peripheral catheterisation (van Oostrom & Knowles, 2018).

There are a number of limitations with this study. The scoring system was previously used in a study examining the application of VS in small animal subjects presenting as a referral or first opinion emergency cases (Trinder et al., 2022). All the score systems used in the present study have not been validated for use in the observed assessment of pain and distress in response to restraint and IC. Of the validated scoring systems in veterinary patients, none were deemed appropriate to assess tolerance to restraint or IC, as they are primarily designed for the assessment of sustained discomfort, and rely on distance examination, with assessment made based on body posture and facial expression (Evangelista et al., 2019).

This study included a heterogenous population of animals as well as intravenous catheter placers, both of which may have significantly influenced patient response. The initial sample size calculation suggested 50 patients per treatment, which was regrettably not obtained for patients receiving EMLA as block randomisation was not employed as a randomisation method. From the initial sample size calculation, a total of 45 patients (the minimum in any group) would achieve 85% power (rather than 90%) and 5% type 1 error rate to adequately detect a four‐fold decrease in OR of response. Given the smaller number of cats in the study, and block randomising for all species and not for each species separately resulted in a significantly greater number consisting of blood donors receiving EMLA, which may have influenced the results. A further study solely of cats, looking at a greater non‐blood donor population may be of benefit.

The use of feline donors may have inadvertently influenced our results given that they are often selected for temperament, and as such may have scored low on reaction scores regardless of the topical anaesthetic used. Similarly, by enrolling oncology patients it may have resulted in a patient population that may have selected for better temperament, as owners of aggressive or extremely nervous patients may be less incline to pursue oncological referral. Additionally, by not including a control group that did not receive any treatment, the study was unable to evaluate the overall efficacy of VS and EMLA and was limited to only comparing the two treatments to each other in this patient population.

The present study demonstrated that there was no significant difference in the response of all patients to IC following the application of ELMA or VS. Additionally, there was no significant difference in the rate of success of first time IC. The efficacy of VS to reduce adverse reaction to skin puncture at the time of IC was significantly less in cats, the present study is underpowered to draw definitive conclusions. Future studies should be used to examine whether differences seen in our study are evident in larger populations.

### Author contributions

**R. Trinder:** Conceptualization (equal); data curation (equal); formal analysis (equal); investigation (equal); methodology (equal); project administration (equal); writing – original draft (equal); writing – review and editing (equal). **J. Park:** Data curation (equal); investigation (equal); methodology (equal); project administration (equal); writing – review and editing (equal). **K. Humm:** Conceptualization (equal); formal analysis (equal); methodology (equal); project administration (equal); writing – review and editing (equal). **L. Cole:** Conceptualization (equal); data curation (equal); formal analysis (equal); investigation (equal); methodology (equal); project administration (equal); writing – review and editing (equal).

### Conflict of interest

None of the authors of this article has a financial or personal relationship with other people or organisations that could inappropriately influence or bias the content of the paper.

## Supporting information


**Table S1.** Reaction scoring for assessment of all animal compliance during restraint and response to their limb being handled, swab application and skin puncture during catheterisation.
**Tablet S2**. Reaction scoring for assessment of all dogs' compliance during restraint and response to their limb being handled, swab application and skin puncture during catheterisation.
**Tablet S3**. Reaction scoring for assessment of all cats' compliance during restraint and response to their limb being handled, swab application and skin puncture during catheterisation.

## Data Availability

The data that support the findings of this study are available on request from the corresponding author. The data are not publicly available due to privacy or ethical restrictions.
