# Supplementary material for: Effectiveness of vapocoolant spray compared to eutectic lidocaine/prilocaine cream to enhance tolerance during intravenous catheterisation: a randomised controlled trial
Source: J Small Anim Pract. 2025 Jan 13;66(4):236–42. doi: 10.1111/jsap.13825 (PMC12000711; doi:10.1111/jsap.13825)
Supplement: Supplementary file 1 — Table S1. Reaction scoring for assessment of all animal compliance during restraint and response to their limb being handled, swab application and skin puncture during catheterisation. Tablet S2. Reaction scoring for assessment of all dogs' compliance during restraint and response to their limb being handled, swab application and skin puncture during catheterisation. Tablet S3. Reaction scoring for assessment of all cats' compliance during restraint and response to their limb being handled, swab application and skin puncture during catheterisation. [file JSAP-66-236-s001.docx]

| Tablet S1: Reaction scoring for assessment of all animal compliance during restraint and response to their limb being handled, swab application and skin puncture during catheterisation | | | | | | | | | | |
| --- | --- | --- | --- | --- | --- | --- | --- | --- | --- | --- |
| Score (0-3) | Behaviour During Restraint:  Struggling | | Behaviour During Restraint:  Aggression | | Reaction to Limb Touch | | Reaction to Swab | | Reaction to IV Catheter | |
|  | VS, n | EMLA, n | VS, n | EMLA, n | VS, n | EMLA, n | VS, n | EMLA, n | VS, n | EMLA, n |
| 0 | 51 | 43 | 56 | 1 | 44 | 40 | 34 | 41 | 30 | 31 |
| 1 | 4 | 1 | 0 | 0 | 3 | 2 | 9 | 2 | 14 | 9 |
| 2 | 1 | 1 | 0 | 0 | 9 | 2 | 10 | 1 | 11 | 3 |
| 3 | 0 | 0 | 0 | 0 | 0 | 1 | 3 | 1 | 1 | 2 |
| EMLA, Eutectic Lidocaine/Prilocaine Anaesthetic; IV, Intravenous VS, Vapocoolant Spray; | | | | | | | | | | |

| Tablet S2: Reaction scoring for assessment of all dogs’ compliance during restraint and response to their limb being handled, swab application and skin puncture during catheterisation | | | | | | | | | | |
| --- | --- | --- | --- | --- | --- | --- | --- | --- | --- | --- |
| Score (0-3) | Behaviour During Restraint:  Struggling | | Behaviour During Restraint:  Aggression | | Reaction to Limb Touch | | Reaction to Swab | | Reaction to IV Catheter | |
|  | VS, n | EMLA, n | VS, n | EMLA, n | VS, n | EMLA, n | VS, n | EMLA, n | VS, n | EMLA, n |
| 0 | 45 | 32 | 50 | 33 | 38 | 29 | 30 | 30 | 29 | 21 |
| 1 | 4 | 0 | 0 | 0 | 3 | 1 | 7 | 2 | 11 | 8 |
| 2 | 1 | 1 | 0 | 0 | 9 | 2 | 10 | 0 | 9 | 3 |
| 3 | 0 | 0 | 0 | 0 | 0 | 1 | 3 | 1 | 1 | 1 |
| EMLA, Eutectic Lidocaine/Prilocaine Anaesthetic; IV, Intravenous VS, Vapocoolant Spray; | | | | | | | | | | |

| Tablet S2: Reaction scoring for assessment of all cats’ compliance during restraint and response to their limb being handled, swab application and skin puncture during catheterisation | | | | | | | | | | |
| --- | --- | --- | --- | --- | --- | --- | --- | --- | --- | --- |
| Score (0-3) | Behaviour During Restraint:  Struggling | | Behaviour During Restraint:  Aggression | | Reaction to Limb Touch | | Reaction to Swab | | Reaction to IV Catheter | |
|  | VS, n | EMLA, n | VS, n | EMLA, n | VS, n | EMLA, n | VS, n | EMLA, n | VS, n | EMLA, n |
| 0 | 6 | 11 | 6 | 11 | 6 | 11 | 4 | 11 | 1 | 10 |
| 1 | 0 | 1 | 0 | 1 | 0 | 1 | 2 | 0 | 3 | 1 |
| 2 | 0 | 0 | 0 | 0 | 0 | 0 | 0 | 1 | 2 | 0 |
| 3 | 0 | 0 | 0 | 0 | 0 | 0 | 0 | 0 | 0 | 1 |
| EMLA, Eutectic Lidocaine/Prilocaine Anaesthetic; IV, Intravenous VS, Vapocoolant Spray; | | | | | | | | | | |
